# Exploring global trends in basic life support (BLS) proficiency: A comprehensive multi-national cross-sectional investigation among medical students and practicing physicians

**DOI:** 10.1097/MD.0000000000051111

**Published:** 2026-10-16

**Authors:** Mohammed Hammad Jaber Amin, Rayan Abdalla Elawad Wedatalla, Azza Omer Osman Abdelrahman, Khadiga Mansoor Ali Bakheit, Maab Khalifa Mohamed Ahmed, Onnab Khalid Alhadi Ahmed, Rayan Mohamed Issa Kheerallah, Sittia Basil Hassan Ali, Mohamed elmusafa Yahya Mohamed Eldouma, Manasik adam ahmed mohamed, Mohammed Adil Ibrahim nouri, Othman Ibrahim Abdullah osman, Nadreen Babikir Mohammad Ibrahim, Abd Elrahman Ahmed Bashir Mohamed, Manahil Tag Uddeen Mohamed Gusm Alseed, Faris Mohamed Abd Allah Mohamed Ahmed, Maha Mujtaba Mahmoud mohamed

**Affiliations:** a Faculty of Medicine, Alzaiem Alazhari University, Khartoum, Sudan; b Medical Resident, Sudan Medical Council, Khartoum, Sudan; c Intern, Maternity and Children Hospital, Dammam, Kingdom of Saudi Arabia; d Medical Student, Ahfad University for Women, Khartoum, Sudan; e Faculty of Medicine, University of Khartoum, Khartoum, Sudan; f Medical Laboratory Science Department, National University of Sudan, Sudan; g Faculty of Medicine, Nile University, Khartoum, Sudan.

**Keywords:** Attitude, Barriers, Basic life support, Knowledge, Medical students, Practice

## Abstract

Basic Life Support (BLS) is integral to emergency medical procedures. Proficiency in BLS is essential for healthcare providers to respond to life-threatening situations effectively. This study aimed to explore the proficiency of BLS practice among medical students and practicing physicians. This cross-sectional study was conducted among medical students and practicing physicians in multiple countries. The data were collected using a questionnaire based on knowledge, attitude, and practice of BLS and barriers to practicing BLS according to the European Resuscitation Council guidelines. In this study, 4478 medical students and physicians participated; most participants were female, 2712 (61%). The respondents were from different ethnic and racial backgrounds. Most respondents, 4102 (92%), know what BLS stands for. More than two-thirds of the participants, 3238 (72%), answered correctly about the compression ventilation ratio. 1666 (37%) participants have never been certified in BLS, and 1384 (31%) claimed they can perform BLS intervention in an emergency. 2844 (64%) strongly agree that access to regular BLS training sessions is a barrier to improving CPR and BLS outcomes. The study reveals adequate knowledge of BLS among medical students and postgraduates. However, BLS practice is poor. A significant number of participants do not acquire adequate practice of BLS. There is an urgent need to encourage medical students and physicians to undergo BLS training.

## 1. Introduction

The Basic Life Support (BLS) program, introduced by the American Heart Association (AHA), serves as the cornerstone for saving individuals experiencing cardiac or respiratory arrest.^[1]^ Maintaining appropriate ventilation and circulation during life-threatening emergencies is a critical medical intervention designed to stabilize patients until paramedics or hospital staff can provide advanced care. BLS techniques include artificial ventilation, bleeding control, cardiopulmonary resuscitation (CPR), and basic airway management.^[2]^ It also involves identifying and treating signs of stroke, heart attack, sudden cardiac arrest, and foreign-body airway obstruction, as well as utilizing an automated external defibrillator (AED) for both CPR and defibrillation.^[3]^

The American Heart Association emphasizes the Chain of Survival as a critical framework for improving cardiac arrest outcomes. This framework comprises prompt recognition of cardiac arrest, activation of the emergency response system, early basic life support (BLS), advanced cardiac life support (ACLS), and comprehensive post-cardiac arrest hospital care.^[4]^ Cardiovascular disease (CVD) remains a significant global health burden, with reports indicating approximately 17.3 million deaths in developing nations attributed to CVD. Furthermore, sudden cardiac arrest accounts for 60% of cardiovascular fatalities, leading to approximately 700,000 injuries annually.

These cardiac events typically occur outside hospital settings and carry extremely low survival rates.^[1]^ Heart arrest is a potentially fatal emergency characterized by an abrupt and unplanned cessation of blood flow when the heart is unable to pump effectively. As a significant cause of death, out-of-hospital cardiac arrest accounts for 0.5 to 1 death per 1000 people annually.^[5]^ However, survival rates can be increased considerably when early BLS with chest compressions is performed before the arrival of an ambulance and advanced life support.^[3]^

Despite the proven efficacy of early BLS intervention, research has revealed that hospital staff members frequently do not possess sufficient knowledge of CPR and BLS protocols.^[6]^ Furthermore, studies indicate that while BLS skills improve markedly with initial training, they deteriorate significantly over time without regular reinforcement.^[3]^ A study conducted in Saudi Arabia revealed that most medical students lack adequate knowledge and readiness for BLS, leading researchers to advise that more BLS instruction and refresher courses be integrated into healthcare college curricula.^[7]^

Given these persistent global knowledge gaps and skill decay patterns, this research attempts to investigate worldwide patterns in medical students’and practitioners’

Basic Life Support knowledge, attitudes, and practices. This comprehensive multi-national investigation aims to pinpoint the specific barriers, disadvantages, and areas where BLS instructions and training can be strengthened internationally and culturally. By comprehending these global trends, we can develop targeted interventions that improve BLS proficiency and, consequently, increase the survival rates of life-threatening emergencies such as cardiac arrest.

## 2. Methodology

### 2.1. Study design, setting, and participants

We conducted a descriptive cross-sectional online survey of medical students and recently graduated physicians from multiple countries using a convenience sampling approach. The electronic survey was published on different social media platforms between April 2 and 19, 2024, and could be accessed by anyone with the link.

Information about the study was shared with a group of collaborators to facilitate data collection. Study information inviting individuals to contribute to the research was disseminated, including the Participant Information Sheet (PIS) and link to the survey. The PIS included information regarding the study’s aims, the protection of participants’ personal data, survey length, and their right to withdraw at any time.

A total of 4478 participants completed the questionnaire. Medical students and recent graduates from several medical schools across the world were approached to participate.

### 2.2. Inclusion and exclusion criteria

Participants were included in the study if they were medical students enrolled in any year of study or practicing physicians, willing to participate in the study, and completed the full questionnaire. Participants were excluded if they had incomplete questionnaire responses, duplicate submissions, were nonmedical participants, or did not provide informed consent.

### 2.3. Data collection tools

An online self-administered questionnaire was developed based on a comprehensive review of existing literature on BLS knowledge, attitudes, and practices, with reference to the European Resuscitation Council guidelines and the American Heart Association BLS guidelines. The questionnaire covered various domains, including:

- **Section 1: Sociodemographic Information** – This section collected data on gender, marital status, highest level of education completed (medical student or postgraduate), residency (urban/rural), employment status, ethnic and racial background, disabilities or impairments, and overall health status.
- **Section 2: BLS Knowledge Assessment** – This section included multiple-choice questions assessing knowledge of the BLS acronym, compression-ventilation ratio, primary goal of chest compression, frequency of BLS certification renewal, and the sequence of BLS steps.
- **Section 3: BLS Practice** – This section assessed BLS certification status, confidence levels, frequency of knowledge updates, AED experience, review of BLS guidelines, and teaching experience.
- **Section 4: Attitude towards BLS** – This section utilized a Likert-scale format (ranging from “Strongly Disagree” to “Strongly Agree”) to assess attitudes toward BLS training, school curricula, public accessibility, refresher training, skill degradation, and employer incentives.
- **Section 5: Barriers to BLS** – This section utilized a Likert-scale format (ranging from “Strongly Disagree” to “Strongly Agree”) to identify barriers to BLS practice, including lack of access to training, limited public awareness, and limited understanding of early defibrillation.

The questions were distributed with randomization to reduce the possibility of response bias, and response validation (completeness check) for all mandatory items was activated to prevent missed answers in the submitted responses. Respondents were able to review and change their answers using a “back button” function.

To ensure questionnaire clarity and relevance, a pilot study involving medical students and recent graduates from diverse academic backgrounds was conducted. The questionnaire items were refined iteratively until adequate clarity and comprehensibility were achieved. Feedback was used to help improve the wording of the initial survey questions, although respondents felt the majority of the questions were clear, relevant, and specific.

### 2.4. Data collection procedure

Data collection was facilitated through Google Forms and distributed by a group of collaborators to personal and professional groups via social media platforms, including Facebook, WhatsApp, Telegram, Twitter, and LinkedIn. Study information was also posted on social media groups for medical students, and reminders to complete the questionnaire were posted. Numerical codes represented categorical data (such as gender and academic year) to facilitate analysis. Respondents’ IP addresses were not collected to maintain anonymity and confidentiality. However, Google Forms only permitted one submission from the same IP address. All personal information was removed to protect participants’ confidentiality.

### 2.5. Statistical analysis

We used the Kolmogorov-Smirnov test to check the normality of the data. To find a significant difference between groups, we used an independent t-test for normally distributed data and the Mann-Whitney test after rejecting the null hypothesis of the Kolmogorov-Smirnov test of normal distribution. We used the chi-square test or Fisher’s exact test to find out if there was a significant difference between the groups for categorical data. A *P*-value equal to or <.05 was considered statistically significant.

### 2.6. Ethical considerations

Ethical approval for this study was obtained from the Federal Ministry of Health of Sudan prior to data collection. The study was conducted in accordance with the ethical principles of the Declaration of Helsinki. All participants were provided with information about the study purpose, the voluntary nature of participation, and data confidentiality measures. Prior to accessing the survey, all potential respondents were presented with an initial disclosure screen outlining the study’s purpose, absolute data confidentiality, and their explicit right to withdraw at any point. Digital informed consent was mandatorily obtained from every participant before data entry was permitted. All data were anonymized to protect participant privacy, and no personally identifiable information was collected or stored. The study adhered to established guidelines for ethical research in medical education.^[8]^

## 3. Results

The primary objectives of this study were to – assess global BLS knowledge and proficiency levels among medical students and practicing physicians, evaluate current BLS certification and training patterns, and identify barriers to BLS skill acquisition and maintenance. A total of 4478 participants completed the questionnaire, with demographic characteristics presented in Table 1.

**Table 1:** Sociodemographic Characteristics of Participants (N = 4478).

Characteristic	Category	n (%)
**Gender**	Female	2712 (61%)
	Male	1740 (39%)
	Prefer not to tell	26 (0.6%)
**Marital status**	Single	3856 (86%)
	Married	586 (13%)
	Divorced	28 (0.6%)
	Widow	8 (0.2%)
**Highest level of education completed**	Medical student	3572 (80%)
	Post Graduate	906 (20%)
**Residency**	Urban	3664 (82%)
	Rural	814 (18%)
**Employment status**	Unemployed	2906 (65%)
	Employed full-time	820 (18%)
	Employed part-time	726 (16%)
	Retired	26 (0.6%)
**Ethnic and racial background**	Black, Asian	1110 (25%)
	African American	748 (17%)
	White/Caucasian	12 (0.3%)
	Pacific Island	6 (0.1%)
	Alaska Native	2 (<0.1%)
	Latino	8 (0.2%)
	Hispanic	2 (<0.1%)
	Native American	4 (<0.1%)
	Other*	2586 (58%)
**Disabilities or impairments**	Yes	240 (5.4%)
**Overall health status**	Poor	58 (1.3%)
	Fair	126 (2.8%)
	Good	928 (21%)
	Very good	1714 (38%)
	Excellent	1652 (37%)

* Other includes respondents who did not identify with the listed racial/ethnic categories or preferred not to specify.

### 3.1. Sociodemographic characteristics

Among the 4478 participants, the majority were female, comprising 2712 (61%) of the sample, while males accounted for 1740 (39%). Married students comprised 586 (13%) of the sample, while single students represented 3856 (86%). Urban areas had a higher response rate, with 3664 (82%) of respondents residing in urban areas compared to 814 (18%) in rural areas. The respondents represented diverse ethnic and racial backgrounds. Regarding employment status, 2906 (65%) of participants were unemployed, while 820 (18%) were employed full-time, and 726 (16%) were employed part-time. Regarding health status, 1714 (38%) of participants reported very good health, 1652 (37%) reported excellent health, and only 240 (5%) reported having disabilities (Table 1).

### 3.2. Knowledge of basic life support

The majority of respondents, 4102 (92%), correctly identified what BLS stands for. More than two-thirds of participants, 3238 (72%), correctly identified the compression-ventilation ratio for adult patients as 30:2. Regarding the primary goal of chest compression, 2418 (54%) correctly identified restoring blood circulation as the primary goal, while only 179 (4%) incorrectly believed restoring consciousness was the primary goal. Fifty-five percent (55%) of participants correctly answered that healthcare providers should renew their BLS certification every 2 years. Regarding the sequence of BLS steps, 2722 (61%) correctly identified the ABC acronym (Airway, Breathing, Circulation) as the sequence used to remember BLS steps (Table 2).

**Table 2:** Knowledge of basic life support among participants (N = 4478).

Question	Response	n (%)
**What does BLS stand for?**	Basic Life Support	4102 (92%)
	Basic Life-saving Skills	158 (3.5%)
	Basic Life Strategy	198 (4.4%)
	Basic Life Synthesis	20 (0.4%)
**What is the correct compression-ventilation ratio for adult patients?**	30:2	3238 (72%)
	15:2	916 (20%)
	5:1	228 (5.1%)
	10:1	96 (2.1%)
**What is the primary goal of chest compression?**	Restoring blood circulation	2418 (54%)
	Restoring consciousness	179 (4%)
	Restoring breathing	1881 (42%)
**How often should healthcare providers renew their BLS certification?**	Every 2 years	2452 (55%)
	Every 5 years	382 (8.5%)
	Every year	1050 (23%)
	Every 3 years	594 (13%)
**What acronym is used to remember the sequence of BLS steps?**	ABC	2722 (61%)
	DRABC	430 (9.6%)
	CPR	672 (15%)
	CAB	654 (15%)

### 3.3. Practice of basic life support

Regarding BLS certification status, 1666 (37%) of participants have never been certified in BLS. Among those certified, 1546 (35%) renew their certification every 2 years, 664 (15%) renew annually, and 602 (13%) renew every 3 years.

Confidence in performing BLS interventions varied among participants. Specifically, 1384 (31%) expressed being somewhat confident, 1214 (27%) reported being very confident, 1024 (23%) were not very confident, and 856 (19%) were not confident at all.

Regarding knowledge and skills updates, 1454 (32%) occasionally review online materials, 1296 (29%) rarely seek updates, 972 (22%) never update their knowledge, and 756 (17%) regularly attend workshops and courses.

Notably, 2168 (48%) of participants have never been trained on automated external defibrillators (AEDs), while 1378 (31%) have received training but never used one. Only 634 (14%) reported successful AED usage, while 298 (6.7%) reported unsuccessful usage.

Regarding review of BLS and CPR guidelines, 2098 (47%) rarely or never review guidelines, while 1010 (23%) review annually, 824 (18%) review semiannually, and 546 (12%) review quarterly.

When asked about teaching BLS courses, 1958 (44%) reported they are not qualified to teach, while 954 (21%) are qualified but do not teach. Among those who teach, 984 (22%) do so occasionally, and 582 (13%) do so frequently (Table 3).

**Table 3:** Practice of basic life support among participants (N = 4478).

Characteristic	Response	n (%)
**BLS certification status**	Never certified	1666 (37%)
	Every 3 years	602 (13%)
	Every 2 years	1546 (35%)
	Every year	664 (15%)
**Confidence to perform BLS interventions in an emergency**	Not confident at all	856 (19%)
	Not very confident	1024 (23%)
	Somewhat confident	1384 (31%)
	Very confident	1214 (27%)
**Frequency of updating BLS, CPR, ACLS, and ATLS knowledge and skills**	Never update	972 (22%)
	Rarely seek updates	1296 (29%)
	Occasionally review online materials	1454 (32%)
	Regularly attend workshops and courses	756 (17%)
**Experience using an automated external defibrillator (AED) in a real-life situation**	No, never trained on AEDs	2168 (48%)
	No, but trained on AEDs	1378 (31%)
	Yes, unsuccessfully	298 (6.7%)
	Yes, successfully	634 (14%)
**Frequency of reviewing BLS and CPR guidelines**	Rarely or never	2098 (47%)
	Annually	1010 (23%)
	Semiannually	824 (18%)
	Quarterly	546 (12%)
**Teaching BLS, CPR, ACLS, or ATLS courses**	No, not qualified to teach	1958 (44%)
	No, but qualified to teach	954 (21%)
	Yes, occasionally	984 (22%)
	Yes, frequently	582 (13%)

### 3.4. The associations of BLS practice with knowledge, attitude, and barrier levels

The analysis showed a statistically significant association between BLS practice level and both knowledge and perceived barriers (both *P* < .001) (Table 4). Participants with moderate BLS practice exhibited the highest proportion of moderate knowledge (66.2%), while those with poor practice were more likely to have poor knowledge (38.5%). Nevertheless, the proportion of participants with good knowledge remained consistently low across all practice groups (approximately 3–5%). In contrast, attitudes toward BLS were uniformly positive, irrespective of practice level. Interestingly, perceived barriers were significantly associated with BLS practice, with participants reporting good practice also exhibiting the highest proportion of maximum barrier levels (68.4%). This seemingly paradoxical finding may indicate that individuals who are more actively engaged in BLS are also more aware of the practical and logistical challenges encountered during training and real-life resuscitation, rather than experiencing fewer barriers.

**Table 4:** The associations of BLS practice with knowledge, attitude, and barrier levels.

Characteristic	Overall, N = 4,478*	Poor, N = 2,046*	Moderate, N = 1,730*	Good, N = 702*	*P*-value†
**Knowledge Levels**					<.001
Poor	1598 (36%)	788 (39%)	502 (29%)	308 (44%)	
Moderate	2702 (60%)	1190 (58%)	1146 (66%)	366 (52%)	
Good	178 (4.0%)	68 (3.3%)	82 (4.7%)	28 (4.0%)	
**Attitude Levels**					.2
Limited	36 (0.8%)	12 (0.6%)	14 (0.8%)	10 (1.4%)	
Partial	100 (2.2%)	42 (2.1%)	38 (2.2%)	20 (2.8%)	
Ample	814 (18%)	386 (19%)	314 (18%)	114 (16%)	
Maximum	3528 (79%)	1606 (78%)	1364 (79%)	558 (79%)	
**Barrier Levels**					<.001
Limited	46 (1.0%)	20 (1.0%)	18 (1.0%)	8 (1.1%)	
Partial	318 (7.1%)	158 (7.7%)	114 (6.6%)	46 (6.6%)	
Ample	1366 (31%)	672 (33%)	526 (30%)	168 (24%)	
Maximum	2748 (61%)	1196 (58%)	1072 (62%)	480 (68%)	

* n (%).

† Pearson’s Chi-squared test.

## 4. Discussion

In this study, we delve into the knowledge, attitude, and practical application of BLS among medical students and practicing physicians. By identifying barriers and gaps, our goal is to enhance BLS training and ultimately improve patient outcomes. BLS proficiency is crucial for healthcare professionals, as it equips them with the necessary tools to save lives in critical situations.^[6]^ In a comprehensive study involving 4478 medical students, researchers investigated BLS awareness and preparedness. The majority of participants were female (61%), reflecting the gender distribution in medical education. Urban areas had a higher response rate (82%) compared to rural regions (18%).^[9]^ Respondents represented diverse ethnic and racial backgrounds. Impressively, 92% correctly identified the acronym “BLS.” Approximately 72% knew the correct compression-ventilation ratio. Notably, 37% had never been certified in BLS, while 31% expressed some confidence in performing BLS interventions during emergencies.^[8,10]^ AED training was lacking, with only 14% reporting successful usage. A significant 64% agreed that limited access to regular BLS training sessions hindered CPR and BLS outcomes. The study revealed that urban inhabitants’ Basic Life Support (BLS) knowledge is better than their rural counterparts. Urban populations typically benefit from higher levels of education and better access to educational resources, including BLS training programs. Conversely, rural populations face challenges related to limited access to educational resources. Poor educational infrastructure and limited opportunities hinder their ability to acquire essential knowledge, including BLS protocols. Urban communities have greater exposure to shareable information through media sources. This exposure likely contributes to their increased familiarity with BLS practices. While urban dwellers can access formal education, rural residents may struggle with poor educational abilities. Literacy rates and effective learning methods play a crucial role in BLS awareness.^[11]^ Numerous studies have consistently shown that trained personnel exhibit better CPR knowledge and skills than untrained individuals.^[12]^ However, retaining these skills remains challenging once they are taught. Post-graduation resuscitation training becomes even more difficult due to busy schedules and limited resources, especially in developing countries. Semeraro et al^[13]^ observed a significant decay in Advanced Life Support (ALS) skills among anesthesiologists 6 months after initial training. Given that guidelines evolve, regular refresher training is essential. Cooper et al^[14]^ reported substantial knowledge and skill enhancement among individuals who had taken a BLS course 6 months prior, especially after a short Intermediate Life Support (ILS) course. Therefore, periodic reinforcement through refresher training is crucial for maintaining adequate CPR skills and continued competency.

This study has several limitations. First, the use of a convenience sampling method via social media may limit the generalizability of the findings. Second, the data is self-reported, which may be subject to recall bias or social desirability bias.

## 5. Conclusion

This study reveals adequate knowledge of BLS among medical students and physicians. Despite having some confidence to perform BLS interventions in emergencies, the majority of them have never been certified or practiced BLS. This issue needs urgent intervention to resolve the barriers to practicing BLS. However, regular training sessions are mandatory to acquire good practical BLS skills. It is recommended that the BLS program should be included in the university curriculum. An organized curriculum for BLS and its protocol-based training are the cornerstones of improving skills among medical students and physicians.

## Author contributions

**Conceptualization:** Mohammed Hammad Jaber Amin, Azza Omer Osman Abdelrahman, Khadiga Mansoor Ali Bakheit, Rayan Mohamed Issa Kheerallah, Mohammed Adil Ibrahim nouri.

**Writing – original draft:** Mohammed Hammad Jaber Amin, Rayan Abdalla Elawad Wedatalla, Azza Omer Osman Abdelrahman, Onnab Khalid Alhadi Ahmed, Sittia Basil Hassan Ali.

**Writing – review & editing:** Mohammed Hammad Jaber Amin, Rayan Abdalla Elawad Wedatalla, Azza Omer Osman Abdelrahman.

**Visualization:** Azza Omer Osman Abdelrahman, Onnab Khalid Alhadi Ahmed.

**Data curation:** Khadiga Mansoor Ali Bakheit, Maab Khalifa Mohamed Ahmed, Rayan Mohamed Issa Kheerallah, Mohamed elmusafa Yahya Mohamed Eldouma, Mohammed Adil Ibrahim nouri, Othman Ibrahim Abdullah osman, Nadreen Babikir Mohammad Ibrahim, Manahil Tag Uddeen Mohamed Gusm Alseed, Faris Mohamed Abd Allah Mohamed Ahmed.

**Formal analysis:** Khadiga Mansoor Ali Bakheit, Mohamed Elmusafa Yahya Mohamed Eldouma, Manasik Adam Ahmed Mohamed.

**Investigation:** Maab Khalifa Mohamed Ahmed, Abd Elrahman Ahmed Bashir Mohamed, Faris Mohamed Abd Allah Mohamed Ahmed.

**Methodology:** Maab Khalifa Mohamed Ahmed, Maha Mujtaba Mahmoud mohamed.

**Resources:** Onnab Khalid Alhadi Ahmed, Manasik Adam Ahmed Mohamed.

**Project administration:** Rayan Mohamed Issa Kheerallah.
